# Adoptively transferred human lung tumor specific cytotoxic T cells can control autologous tumor growth and shape tumor phenotype in a SCID mouse xenograft model

**DOI:** 10.1186/1479-5876-5-29

**Published:** 2007-06-25

**Authors:** Ezogelin Oflazoglu, Mark Elliott, Hiroshi Takita, Soldano Ferrone, Robert A Henderson, Elizabeth A Repasky

**Affiliations:** 1 Department of Immunology Roswell Park Cancer Institute, Buffalo, NY, 14263 USA; 2 Department of Surgery, Roswell Park Cancer Institute, Buffalo, NY, 14263 USA; 3 Corixa Corporation, Seattle, WA, 98101 USA

## Abstract

**Background:**

The anti-tumor efficacy of human immune effector cells, such as cytolytic T lymphocytes (CTLs), has been difficult to study in lung cancer patients in the clinical setting. Improved experimental models for the study of lung tumor-immune cell interaction as well as for evaluating the efficacy of adoptive transfer of immune effector cells are needed.

**Methods:**

To address questions related to the *in vivo *interaction of human lung tumor cells and immune effector cells, we obtained an HLA class I ^+ ^lung tumor cell line from a fresh surgical specimen, and using the infiltrating immune cells, isolated and characterized tumor antigen-specific, CD8^+ ^CTLs. We then established a SCID mouse-human tumor xenograft model with the tumor cell line and used it to study the function of the autologous CTLs provided via adoptive transfer.

**Results:**

The tumor antigen specific CTLs isolated from the tumor were found to have an activated memory phenotype and able to kill tumor cells in an antigen specific manner *in vitro*. Additionally, the tumor antigen-specific CTLs were fully capable of homing to and killing autologous tumors *in vivo*, and expressing IFN-γ, each in an antigen-dependent manner. A single injection of these CTLs was able to provide significant but temporary control of the growth of autologous tumors *in vivo *without the need for IL-2. The timing of injection of CTLs played an essential role in the outcome of tumor growth control. Moreover, immunohistochemical analysis of surviving tumor cells following CTL treatment indicated that the surviving tumor cells expressed reduced MHC class I antigens on their surface.

**Conclusion:**

These studies confirm and extend previous studies and provide additional information regarding the characteristics of CTLs which can be found within a patient's tumor. Moreover, the *in vivo *model described here provides a unique window for observing events that may also occur in patients undergoing adoptive cellular immunotherapy as effector cells seek and destroy areas of tumor growth and for testing strategies to improve clinical effectiveness.

## Background

Lung cancer is relatively resistant to currently available chemotherapy and radiotherapy regimens. However, observations of lymphocytic infiltrates or generation of cytotoxic T lymphocytes (CTLs) recognizing lung tumor antigens in murine and human tumors [1-5] suggest that an immune reaction could potentially help eliminate tumor cells. While immune effector cells are present, often in high numbers, immunological rejection of tumors is usually not complete, indicating that there may be defects in the generation or execution of an anti-tumor immune response [6]. Many factors likely contribute to an incomplete immune response including defective processing and presentation of tumor antigens and loss of MHC class I antigen expression [7], absence of co-stimulation [8], absence of tumor reactive T cells [9,10], alteration in T cell signaling [11-14], presence of a variety of immunosuppressive factors [15,16], and inhibition of tumor T cell infiltration [17,18]. Clearly the dynamics between a growing tumor and host is very complex and cannot be explained by a single mechanism. Most of these mechanisms are currently studied in murine tumor models. There are relatively few reports about the actual events that occur *in situ *between human lung tumors and effector cells of the immune response.

Since the major biological activity against tumors *in vitro *and *in vivo *displayed by tumor infiltrating lymphocytes (TILs) can be attributed to CD8^+ ^T cells [19], adoptive immunotherapy using *in vitro *expanded tumor antigen-specific CD8^+ ^CTLs, has been considered as a feasible option for *in vivo *eradication of tumors [20]. A variety of lymphoid cells that are able to lyse tumor cells have been described [19-24]. Furthermore, in humans the *in vitro *generation and expansion of autologous CTLs that recognize lung [1-5], melanoma [25-27], renal [28], ovarian [29], leukemia [30-32], lymphoma [33,34] and breast [35-38] tumor antigens have now been reported. Since tumor antigen-specific CTLs are effector cells that are known to play an important role in inhibiting tumor growth, it is important to understand the interaction between tumor-specific CTLs and autologous lung tumors. Although there were subcutaneous models that generated to study these interactions [5], it is also important to examine the interaction *in vivo *between these cells and their target tumor grown orthotopically. The difficulties in studying the interplay between a patient's immune response and tumors highlights the need to develop an *in vivo *model to study the interaction between these cells in a model which may replicate more closely their environment in patients.

In this study, primary lung tumor cells and tumor antigen-specific CTLs isolated from the original surgical specimen of that same patient's tumor were used as the target and effector cell, respectively. We demonstrate that the CTLs were capable of selective homing to and killing autologous lung tumors grown either subcutaneously or as experimental metatastases in the lung of SCID mice. We also observed that there was a defined period of time after a single injection of CTLs in which the tumor's growth was inhibited and after this point the tumors regrew. Most importantly, the MHC class I antigen expression on the surviving tumor cells was significantly reduced or absent compared to the original tumor. These data and the use of this model for development of improved adoptive T-cell based immunotherapeutic approaches to cancer are discussed.

## Methods

### Animals

Six to eight week old male CB17-scid/scid mice used for this study were obtained from Taconic Labs (Germantown, NY). They were housed in microfilter cages (Lab Products, Maywood, NJ) at Roswell Park Cancer Institute. All cages, water, and food (Teklad Mills, Wienfield, NJ) were supplied after autoclaved. The cages were maintained in an air-conditioned and light-controlled (12 h/day) room and all handling and operations were done in a laminar flow hood.

### Tumor cells

The cell line LT 391-06 was derived from a solid tumor mass excised from the lung of a patient with large cell lung carcinoma. The tumor was obtained as a portion of a surgical specimen, minced with scalpels and digested for 4 hours at 37°C in AIM-V (GibcoBRL, Carlsbad, CA) containing Dispase 8 ug/ml (GibcoBRL) Collagenase 100 ug/ml (GibcoBRL) and Dnase, 10 Ku/ml (Roche). The resultant cell slurry was passed through a nylon mesh screen to break up clumps and layered over two densities of Lymphoprep (Axis-Shield, Oslo, Norway). 7 ml of 1.077 density Lymphoprep was added to a 15 ml conical centrifuge tube followed by 7 ml of a 75% Lymphoprep, 25% 1 × PBS mixture. 10 ml of the cell slurry was layered atop the Lymphoprep and was separated by density centrifugation. The layers were collected sequentially with the top layer containing the majority of tumor cells and the lower layer containing the majority of TIL. Tumor was resuspended and cultured in a mixture of 50% F12 nutrient mixture (GibcoBRL) 50% Basal Medium Eagle (GibcoBRL) 10% FBS (HyClone, Logan, UT) 100 u/ml Penicillin, 100 ug/ml streptomycin, 2 mM L-glutamine (GibcoBRL) 5 × 10^5 ^β-mercaptoethanol. Tumor cells were incubated at 37°C and carefully monitored for growth and expansion. Immunohistochemistry (IHC) was performed by Phenopath Laboratories, Seattle, WA in order to confirm that expanding cell lines indeed arose from a lung carcinoma. This tumor cell line expresses HLA-A11, B44 and Cw1203. The lung tumor cell line 936T derived from a surgically removed squamous lung carcinoma lesion and the lung tumor cell line 81-86T derived from a surgically removed large cell carcinoma lesion were kindly provided by Dr. Jill Siegfried of the University of Pittsburgh, Pittsburgh, PA. The 936T cell line expresses the same tumor antigen that is expressed by the LT-391 06 cell line, and expresses HLA-Cw1203 antigen. The cell line 81–86 expresses the tumor antigen but has a HLA phenotype different from that of the cell line LT391-06. 81-86-1203 is the lung tumor cell line 81–86 transfected with HLA-Cw1203 used for positive control. HLA-Cw1203 stable transfectants of tumor cells were generated by retroviral transfection resistant to blasticidin enabling drug selection. A549 is an allogeneic bronchioalveolar carcinoma cell line purchased from ATCC (Cat# CCL 185). This tumor cell line is antigen negative and expresses HLA B44 and Cw1203 antigens. HLA typing of tumor cells was performed at the Puget Sound Blood Center in Seattle, WA. Tumor cells were maintained at 37°C in a 5% CO_2 _atmosphere in BME/Nutrient mixture F-12 (Life Technologies, Inc., Grand Island, NY), supplemented with 5% heat-inactivated FBS. Cells were allowed to grow to 80% confluency and were harvested with 0.25% typsin-1 mM EDTA in HBSS.

### Cytotoxic T lymphocytes

Tumor infiltrating lymphocytes (TIL) that the CTLs derived from were expanded post extraction from the tumor matrix (described above) and cultured in RPMI 1640 medium (GibcoBRL)containing 10% heat inactivated human serum obtained from an in house donor pool, 100 u/ml Penicillin, 100 ug/ml streptomycin, 2 mM L-glutamine (GibcoBRL). The media was supplemented with 100 u/ml Proleukin IL-2 from (Chiron Therapeutics, Seattle, WA) and 30 ng/ml Orthoclone OKT3 (Ortho Biotech). Rapidly growing T cells were split upon confluence and re-fed with fresh media containing a final concentration of 100 u/ml IL-2 and 30 ng/ml mAb OKT3. Tumor antigen-specific T cell lines were obtained by stimulating 1.5 × 10^6 ^T-cells with 1 × 10^5 ^gamma irradiated (20000 rads) autologous tumor cells over a 7-day cycle. The stimulations were performed in 24 well plates, in 2 ml CTL medium, containing 25 μ/ml IL-2, 10 ng/ml IL-7 (Endogen, Woburn, MA). Media was replaced every 2 – 3 days with IL-2 and IL-7. T cell lines were tested for specificity in a chromium release assay. T cell lines with specific cytotoxicity were cloned by limiting dilution. Per well, T cells, 1 × 10^4 ^allogeneic irradiated LCL (8000 rads), 7.5 × 10^4 ^allogeneic irradiated PBL (4000 rads) 50 u/ml IL-2 and 30 ng/ml mAb OKT3, 200 ul CTL media per well. Growth positive wells were tested for specificity in a 5 hour chromium release assay. Responding wells were expanded in T25 flasks containing 20 ml CTL media, 50 μ/ml IL-2, 30 ng/ml mAb OKT3, 25 × 10^6 ^irradiated allogeneic PBMC (4000 rads), 5 × 10^6 ^allogeneic LCL (8000 rads) and approximately 2 × 10^5 ^of the specific clone. V-beta analysis performed upon isolated clones by flow cytometry confirmed the homogeneity of the clone. Expanded cells were viably frozen in 90% Fetal Calf Serum (HyClone, Logan, UT) and 10% DMSO (Sigma Chemical co, St Louis, MO). Cells were thawed and rested 24 hours in CTL media containing 25 μ/ml IL-2 prior to each use. Anti-LT391-06 CTL is tumor antigen specific CD8^+ ^T cell clone derived from TILs of lung tumor patient number LT391-06. This clone recognizes a unique shared antigen restricted by HLA-Cw1203 antigen. Studies to identify more precisely this antigen are currently in progress. A CD8^+ ^T cell clone which recognizes an influenza nucleoprotein specific antigen restricted by HLA-B27 antigen was used as a control. Cells were cultured at 37°C in a 5% CO_2 _atmosphere in RPMI 1640 medium (Life Technologies, Inc.) supplemented with 10% heat-inactivated FBS.

### Antibodies

Antibodies for phenotyping the cell surface of CTLs included mouse anti human CD2, CD3, CD4, CD8, CD11a (LFA-1), CD25 (IL-2Rα), CD27, CD28, CD29 (β1-integrin), CD44, CD45RO, CD45RA, CD49a (α1 integrin), CD49b (α2 integrin), CD49d (α4 integrin), CD49e (α5 integrin), CD49f (α6 integrin), β7-integrin, CD54 (ICAM), CD56 (NCAM), CD62L (L-selectin), CD69, CD95 (Fas), CD95L (FasL), CD122 (IL-2Rβ), CD132 (IL-2Rγc), CD134 (OX40), CD152 (CTLA-4), CD154 (CD40L), CD161 (NK1.1), IL-6R, IL-9R, IL-10R, CCR5, CCR7, CXCR3 and MHC Class 1 antigens. All these antibodies were purchased from BD-Pharmingen (San Diego, CA). The mAb HC10 which recognizes a determinant expressed on all β_2_-microglobulin-free HLA-B and HLA-C heavy chains, as well as on β_2_-microglobulin-free HLA-A10, HLA-A28, HLA-A29, HLA-A30, HLA-A31, HLA-A32, and HLA-A33 heavy chains was developed and characterized as described [39,40].

### Flow cytometry

Single color staining was performed by incubating 5 × 10^5 ^viable cells with an optimal amount of fluorescence-coupled antibodies. After several washes, approximately 10,000 cells per sample were analyzed on a FACScan (Beckton Dickinson, San Jose, CA).

### Cytokine measurement

Supernatant cytokine release was determined by sandwich ELISA. Serial dilutions of samples were analyzed for IFN-γ and TNF-α. Antibodies were purchased from BD-Pharmingen.

### Chromium release assay

Tumor cells were labeled with 3.7 MBq (100 μci) ^51^Cr for 1 hr at 37°C. Tumor cells were washed and re-suspended in RPMI 1640 medium supplemented with 10% FBS. After counting, CTLs were placed in 96 well plates. Subsequently ^51^Cr labeled tumors cells were added to appropriate wells. Following a 5 hr incubation at 37°C in a 5% CO_2 _atmosphere, plates were centrifuged at 400 rpm for 5 min. The supernatant of each well was collected individually and the ^51^Cr release was assessed in a gamma counter. The percentage of specific ^51^Cr release was calculated using the standard formula: %^51^Cr release = [(^51^Cr release of samples-spontaneous ^51^Cr release)/(maximum ^51^Cr release-spontaneous ^51^Cr release)] ×100. All samples were run in quadruplicate in two different sets of experiments.

### Tumor growth

One hundred μl of a lung tumor cell suspension (at a concentration of 5 × 10^6^/ml) were injected subcutaneously or intravenously into SCID mice. Subcutaneously grown tumors were measured with a venier caliper to determine the shortest diameter (A) and the longest diameter (B). The volume was then calculated by using the formula V = (A^2^B)/2. For intravenously grown tumor, lung were excised and weighed.

### Depletion of murine NK cells

To deplete NK cell activity, 24 h before injection of tumor cells, we injected every 4 weeks i.p 100 μl of TMβ-1 antibody obtained as ascites fluid from a hybridoma kindly provided to Dr. Richard Bankert (State University of New York at Buffalo, Buffalo, NY) by Dr. T. Tanaka (Tokyo Metropolitan Institute of Medical Science, Tokyo, Japan). This antibody blocks murine IL-2β receptor and exhibits no crossreactivity with human IL-2β receptor [41].

### IL-2 treatment

Subcutaneous tumor bearing SCID mice, previously treated with TM-β1 antibody, received daily subcutaneous injections of 20,000 IU of Proleukin IL-2 (Chiron Therapeutics, Seattle, WA) starting the day of adoptive transfer of CTLs.

### Histopathology

After animals were sacrificed, tumor and lung or other organs were excised for histological analysis. Excised tissues were fixed for 24 h in 10% buffered formalin. The tissues were then dehydrated for 18 h by an automatic tissue processor and embedded in paraffin blocks. Five-micrometer paraffin tissue sections were cut and the tissue sections were stained with hematoxylin and eosin.

### Immunohistochemical analysis

Immunohistochemical analysis was performed according to the staining procedure for paraffin sections provided by Vector Laboratories (Burlingame, CA). Briefly, paraffin sections (5 μm) were deparaffinized and rehydrated through xylene and alcohol series. The tissue sections were then incubated with 0.3% H_2_O_2 _in methanol for 30 min to quench endogenous peroxidase activity and then incubated with diluted normal goat serum for 20 min to block nonspecific bindings. The sections were then incubated at room temperature for an hour with anti-HLA class I heavy chain mAb HC-10 or CD8 (Novocastra Laboratories Ltd., Newcastle, UK). Biotinylated goat anti-mouse IgG antibodies (at a1:500 dilution) were then applied to tissue sections for 30 min at room temperature. Vectorastain ABC kit (Vector Laboratories, Burlingame, CA) was used for signal enhancement and peroxidase substrate DAB was used for color development.

### RT-PCR

Total RNA was extracted from lung and liver of SCID mice baring tumor and/or CTL and RT-PCR was done as per manufacturer's instructions (Superscript Preamplification System; Life Technologies, Inc). Primers for human CD8 were: 5'-TTTAGCCTCCCCCTTTGTAAAACGGGCG-3' and 5'-TTTCGGCGAGATACGTCTAACCCTGTGC-3'. Primers for human IFN-γ were: 5'-CTCCTTTTTCGCTTCCCTGTTTTAGCTGCTGG-3' and 5'-GCATCGTTTTGGGTTCTCTTGGCTGTTACTGC-3'. Primers for β-actin were: 5'-ATCTGGCACACCTTCTACAATGAGCTGCG-3' and 5'-CGTCATACTCCTGCTTGCTGATCCACATCTGC-3'. Amplification was done for 35 cycles with denaturing at 94°C for 30 s, annealing at 60°C for 1.5 min, and extension at 72°C for 1.5 min.

### Statistical analysis

Statistical analysis was conducted using two methods. For lung weights we used non-parametrical unpaired Student t-test analysis. For analysis of sc models we took the reference as the day for tumors to reach 500 mm^3 ^in volume using the long rank test provided in the Graphpad Prism Software Package version 4.01 (Graphpad, San Diego, CA). P values < 0.05 referred as statistically significant.

## Results

### Patient derived CTLs express an activated memory phenotype and kill autologous lung tumor cells in vitro

When patients undergo CTL adoptive therapy, CTLs are usually obtained from the tumor and expanded *in vitro *to obtain a sufficient number of cells. To investigate parameters associated with adoptive immunotherapy of a patient's CTLs, we used a SCID mouse model bearing the autologous lung tumor. Briefly, we used the TILs derived from a patient's lung tumor to obtain a population of CTLs for expansion. We first characterized the cell surface markers of the CTLs that we expanded *in vitro*. Our analysis revealed that these expanded CTLs have an activated memory phenotype and maintain the phenotype of the CTLs originally isolated TILs: CD3^+ ^CD8^+ ^CD25^+ ^CD27^- ^CD44^+ ^CD69^+^CD62L^-^CD45RA^- ^CD45RO^+ ^CCR5^+ ^CCR7^+^.

**Figure 1:**
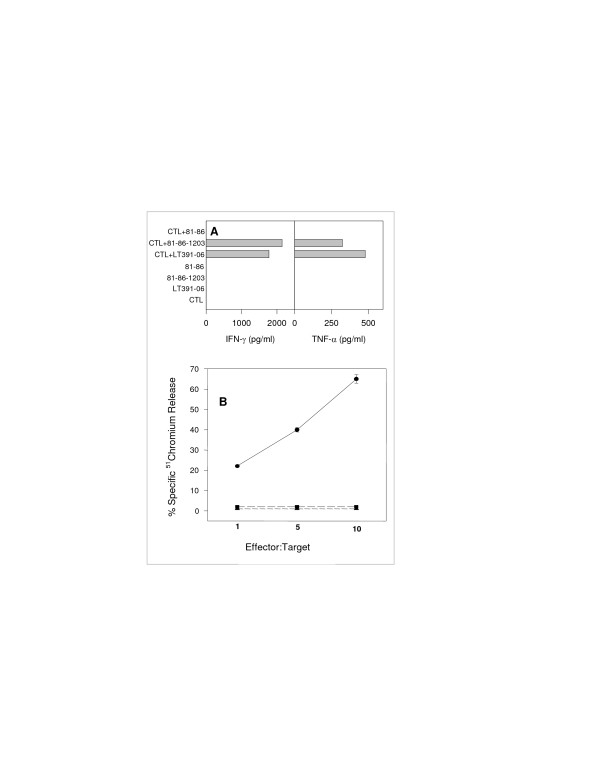
Tumor antigen-specific CTLs release cytokines and exert cytotoxic activity when incubated *in vitro *with autologous tumor cells. **A**. CTLs (1 × 10^4^) were incubated with an equal number of 81–86 (allogeneic tumor, a negative control), 81-86-1203 tumor cells (allogeneic tumor transfected with HLA-Cw1203, a positive control), or LT-391-06 tumor cells (autologous tumor). CTLs and tumor cells were also incubated alone as controls. Cells were incubated overnight at 37°C. Supernatants were analyzed by sandwich ELISA for the presence of IFN-γ and TNF-α. **B**. The cytotoxic activity of CTLs was analyzed by a ^51^chromium release assay. Serial dilutions of CTLs (starting from 1 × 10^4^) were incubated with either (1 × 10^3^) LT-391-06 (●) or Daudi (▲, negative control) or K562 (■, negative control).

In order to determine whether these HLA-Cw1203 restricted CTLs were capable of recognizing and responding to tumor cells, they were co-cultured with tumor cells that either expressed or did not express the appropriate restriction element. In the presence of autologous tumor cells these CTLs produced IFN-γ and TNF-α in tissue culture supernatant (Fig 1A) and were able to specifically lyse tumor cells *in vitro *as demonstrated in a chromium release assay (Fig 1B). CTLs were neither able to produce IFN-γ and TNF-α nor lyse tumor cells that lacked the appropriate restriction element (Fig 1A, B).

### Tumor infiltration by tumor antigen-specific CTLs

**Figure 2:**
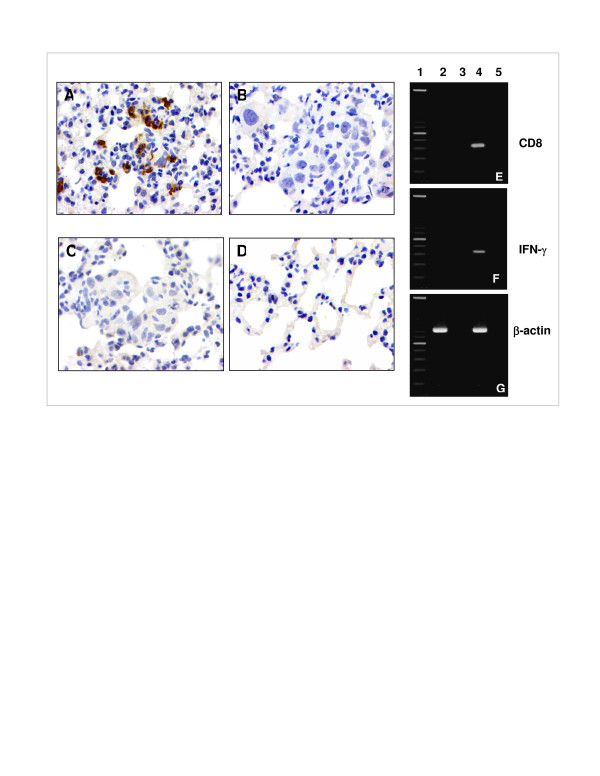
Tumor antigen-specific CTL and expression of cytokines are detected in the lungs of SCID mice bearing autologous lung tumor. Mice were injected with 5 × 10^5 ^tumor cells intravenously. On day 5, after tumor foci were established in the lungs, 5 × 10^6 ^CTLs were injected intravenously. On day 10, mice were sacrificed and lung and liver were excised for immunohistochemical and PCR analysis. **A, B, C **and **D**. IHC analysis of lungs for the presence of CD8 positive CTLs. Lungs from mice injected with **A**. tumor cells + tumor antigen-specific CTLs **B**. tumor cells only, **C**. tumor cells + influenza nucleoprotein antigen-specific CTL or **D**. tumor antigen-specific CTL alone. Original magnification: ×200, brown staining shows CD8 positive T cells. **E, F **and **G**. RT-PCR analysis. Primers used are specific for human **E**. CD8, **F**. IFN-γ and **G**. β-actin. *Lane 1*, 100 bp DNA ladder; *lane 2*, cDNA from lung of SCID mice bearing human tumor only; *lane 3*, cDNA from liver of SCID mice bearing with tumor only; *lane 4*, cDNA from lung of SCID mice bearing human tumor and CTLs; *lane 5*, cDNA from liver of SCID mice bearing tumor and CTLs.

We next investigated the ability of these CTLs to localize at the site of orthotopically grown autologous lung tumor cells in SCID mice. In preliminary experiments, we found that when these tumor cells were injected intravenously they seeded to the lungs and formed established tumor foci by day 5 (data not shown). Therefore, we chose to adoptively transfer CTLs via tail vein injection at day 5. Lungs and other organs were recovered from mice at day 10 and examined for the presence of CD8 ^+ ^CTLs by immunohistochemistry and RT-PCR analysis. We found that CTLs homed and persisted only to the tumor bed in the lungs (Fig 2A and 2E) but were not found in the liver (Fig 2E), as determined not only by microscopic analysis of multiple sections (data not shown) but also by the complete lack of human β-actin expression in liver (Fig 2G). Immunohistochemical analysis demonstrated that while tumor antigen-specific CD8^+ ^CTLs injected on day 5 localized and persisted within the lung tumor at day 10 (Fig. 2A), flu-specific CTLs were not found in the lung tumor examined on the same day (Fig 2C). As expected, the lungs of the mice that were injected with only tumor cells but not CTLs as a control did not stain for the CD8 marker (Fig 2B and 2E). CD8+ CTLs did not accumulate in the lungs of non-tumor bearing mice (Fig 2D). These results indicate a requirement for the presence of an appropriate antigen at the site of the lung for sustaining the presence of the reactive CTLs in this site. We observed human IFN-γ expression in lungs bearing both autologous tumors and tumor antigen-specific CTLs, but not in lungs bearing tumor only (Fig 2F). These data show that a lung tumor cell line derived from a patient's lung tumor can be tumorigenic and colonize the lung of SCID mice. In addition, autologous tumor antigen-specific CTLs can home to and persist in these established tumors. Moreover, specific production of cytokines from tumor antigen-specific CTLs is consistent with the conclusion that the autologous tumor cells continued to express the tested tumor antigen when grown as xenografts. The ability of CTLs to express IFN-γ *only *in the presence of tumor suggests that the CTLs remain functional following adoptive transfer to SCID mice.

### Anti-tumor effect of CTLs on established lung tumors in vivo

**Figure 3:**
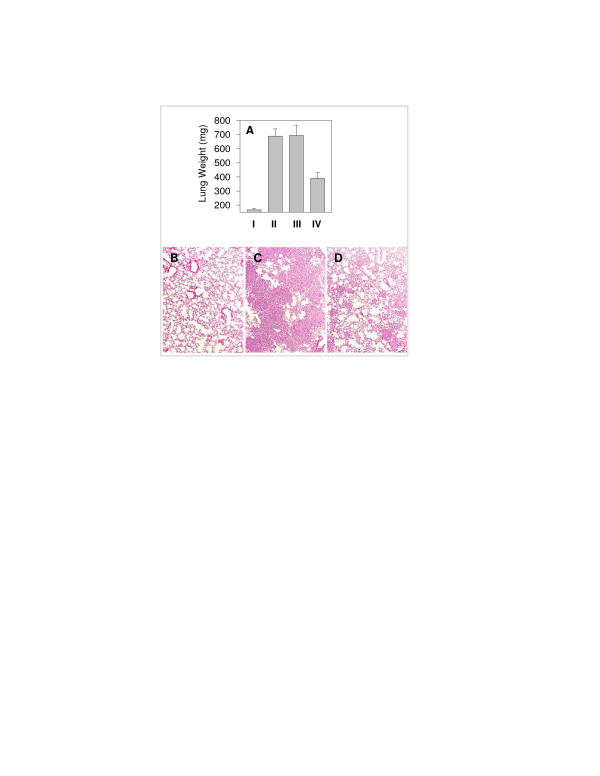
Tumor specific CTLs suppress the growth of autologous lung tumors in SCID mice. SCID mice were injected with 5 × 10^5 ^tumor cells intravenously. At day 5, after tumor foci were established in the lungs, mice were received either 5 × 10^6 ^tumor specific or anti-flu CTLs (i.v. injection). At day 20 and 35, 6 mice from each group (I. control; II. tumor only; III. tumor plus flu-specific CTL or IV. tumor plus tumor-specific CTL were sacrificed and lung and liver were excised for further studies. **A**. Lung weights; **B**. Histology of normal lung; **C**. Histology of lung from tumor bearing mice treated with anti-flu specific CTLs and **D**. Histology of lung from tumor bearing mice treated with tumor specific anti-LT391-06 CTLs. Differences in lung weight between groups was evaluated by the student t-test: II versus III, *P *< 0.95; II vs. IV, *P *< 0.001; III vs. IV, P < 0.004.

**Figure 4:**
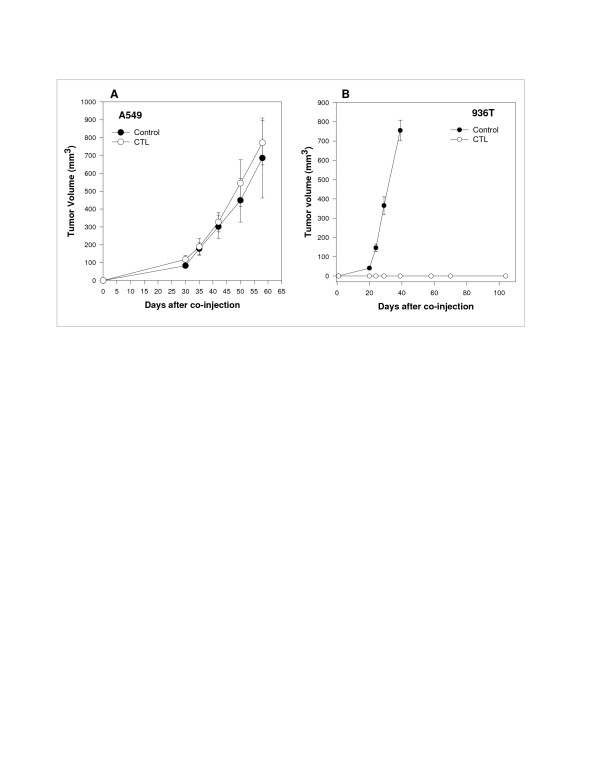
The anti-tumor effect of anti-LT391-06 CTLs against two HLA matched allogeneic tumor cells is antigen specific. SCID mice in control groups (●) received a s.c. injection of 5 × 10^5 ^tumor cells alone and mice in the treated groups (○) received a co-injection of 1 × 10^6 ^anti-LT391-06 CTLs s.c. **A**. Mice bearing A549 tumors, an allogeneic bronchioalveolar carcinoma cell line which does not express the LT391-06 Ag recognized by the tumor specific CTLs; **B**. Mice bearing 936T, an allogeneic tumor cell line derived from a squamous lung carcinoma which does express this antigen. (*n *= 5 mice/group)

To determine whether tumor antigen-specific CTLs could inhibit tumor growth *in vivo *we injected mice with autologous tumor cells on day 0 and CTLs on day 5 intravenously. On day 35 lungs were removed and based on lung weight measurements (Fig. 3A) and histological analysis (Fig. 3B), we found that a single dose of CTLs was effective in suppressing the growth of lung tumors. To confirm that this CTL-mediated tumor suppression is tumor antigen-specific *in vivo*, we used two allogeneic cell lines as controls. 936T lung tumor cells shared the same antigen and expressed HLA-Cw1203 whereas A549 tumor cells did not. These tumor cells were each co-injected with LT391-06-specific CTLs. LT391-06-specific CTLs inhibited *in vivo *the growth of HLA-matched allogeneic cells expressing antigen 936T (Fig 4B), but had no detectable effect on the growth of HLA-matched allogeneic tumor cells lacking antigen A549 (Fig 4A).

### Effect of timing and frequency of CTL injections on established lung tumor growth

**Figure 5:**
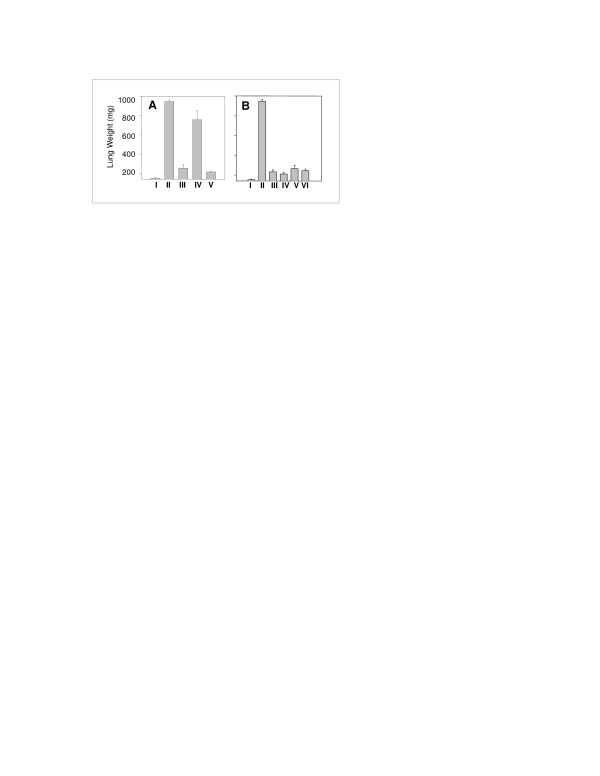
The anti-tumor effect of CTLs on autologous tumor xenografts is dependent on the time of administration, but not the number of doses. **A**. Mice (5/group) received 5 × 10^5 ^tumor cells (i.v.). Once tumors were established in the lung, three of these groups (5 mice/group) were injected with CTLs (5 × 10^6^): Group III on day 5, Group IV on day 15 and Group V on both days 5 and 15. (The control group I did not receive tumor, Group II received tumor but not CTLs). At day 30, mice were sacrificed and lungs were excised and weighed. Differences in lung weight between treatment groups were evaluated by the Student t-test: I vs. II, *P *< 0.001; I vs. III, *P *< 0.1; I vs. IV, *P *< 0.001; II vs. IV, P < 0.35; III vs. IV, P < 0.001. **B**. Mice (groups II-IV, 5 mice/group) were injected with 5 × 10^5 ^tumor cells (i.v.). After lung tumors were established, 4 groups (III-VI) received CTLs (5 × 10^6^) as follows: Group III – one dose of CTL on day 3; Group IV – days 3 and 4; Group V-days 3, 4 and 5; Group VI-days 3, 4, 5 and 6. Normal lung weight was derived from Group I, which did not receive tumor cells; Group II received PBS and no CTL. On day 30, mice were sacrificed and lungs were excised and weighed. The growth of lung tumors in mice treated with tumor specific CTLs was significantly inhibited by one dose on day 3; there was no advantage to the administration of additional CTL doses.

Next, we investigated whether additional injections or different schedules of CTL injection could improve the efficacy of tumor suppression. Figure 5 compares the lung weights of control animals (Group 1) with animals injected with tumor only (Group 2). In figure 5A tumor bearing animals were subjected to CTL injection either on day 5 (Group III) or on day 15 (Group IV) or both on days 5 and 15 (Group V). Based on these lung weight measurements on day 30, we found that administration of CTLs on day 5 resulted in a significant inhibition of tumor growth in comparison to administration of CTLs on day 15; in the latter case CTLs had no significant effect on tumor growth. In addition, injection of CTLs on both day 5 and 15 did not significantly enhance the anti-tumor effect in comparison with CTLs injected only on day 5. These results indicate that the timing of CTL injection is critical for suppression of tumor growth (Fig 5A). Since, two injections of CTLs spaced 10 days apart were not more effective than one injection on day 5, we specifically investigated the effect of frequency of CTL injection on antitumor activity. One dose of CTLs on Day 3 resulted in significant tumor growth inhibition which was not enhanced by additional doses on successive days (Fig 5B). We also examined the effect of daily injection of 20,000 IU of IL-2 on antitumor activity of CTLs and found that injection of IL-2 for three weeks did not enhance their antitumor activity (data not shown).

### Decrease in surface HLA class I expression in lung tumor treated with tumor antigen-specific CTLs

**Figure 6:**
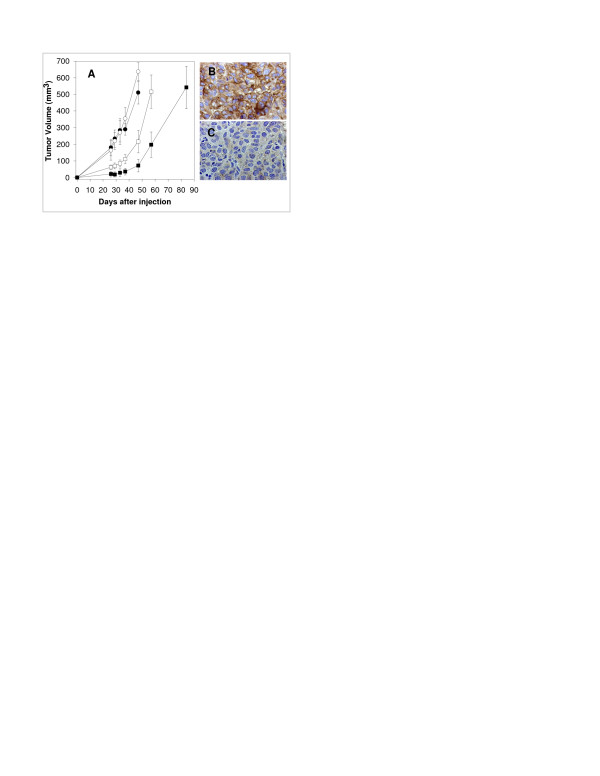
Human tumor growth inhibition in SCID mice treated with CTLs. **A**. Four groups of SCID mice (5 mice/group) were used. Group I (●) received LT391-06 tumor cells only (s.c. injection of 5 × 10^5 ^cells); Group II (○) received tumor cells and 1 × 10^6 ^influenza specific CTLs (nucleoprotein specific, HLA-B27 restricted); Group III (□) received 5 × 10^5 ^tumor cells and 5 × 10^5 ^tumor specific anti-LT391-06 CTLs and Group IV (■) received 5 × 10^5 ^tumor cells and 1 × 10^6 ^tumor specific anti-LT391-06 CTLs. When the tumor volume reached ~500 mm^3^, mice were sacrificed and the tumors were excised for further histological studies. The graph shows means ± s.d. of tumor volume. **B, C**. HLA class I antigen expression in lung tumor cells grown subcutaneously with CTL **B**. Subcutaneously grown human lung tumor cells co-injected with influenza specific CTL clone or **C**. subcutaneously grown human lung tumor cells co-injected with tumor antigen specific CTL clone in mice. Tumor tissues were fixed in formalin and stained with HLA class I heavy chain-specific mAb HC-10. Original magnification: ×400

Tumor growth in the SCID mouse model utilized is suppressed by tumor antigen-specific CTL in a dose dependent fashion for approximately 40 days (Fig 6A). After this time interval, the growth rate of tumors undergoes a rapid acceleration. Since defects in HLA class I antigen expression have been identified as an escape mechanism underlying tumor progression in spite of a tumor antigen-specific CTL response, we asked whether tumors which continued to progressed after treatment with CTLs underwent alteration of HLA class I expression. As shown in Fig 6C, HLA class I expression is significantly reduced in tumor cells grown in the presence of tumor antigen-specific CTLs. The effect is specific since no significant change in HLA class I expression was detected in the presence of influenza specific CTL (Fig 6A). These results were notable because the tumor cells that we prepared for these experiments were originally positive for HLA class I expression *in vitro *and remained positive by IHC in untreated mice (data not shown).

## Discussion

Despite theoretical appeal and actual data regarding the efficacy of T cell-mediated immunotherapy, only a small subset of human cancer patients who present with tumors actually experience clinically relevant benefits. The results of our studies presented here indicate that tumor antigen-specific, HLA-Cw1203 restricted CTLs, generated from the cells that infiltrate a patient's lung tumor, are able to lyse autologous lung tumor cells *in vitro *and in a SCID mouse model. Consistent with these findings, in humans the *in vitro *generation and expansion of autologous CTLs that recognize lung [1-5], melanoma [25-27], renal [28], ovarian [29], leukemia [30-32], lymphoma [33,34] and breast [35-38] tumor antigens have been reported. Furthermore, studies using patients' melanoma tumors [42,43] or revealed that melanoma antigen-specific CTLs killed allogeneic melanoma cells which shared the HLA class I restricting element, to a similar extent as autologous melanoma cells. Other studies showed that CTLs can be effective cytotoxic effector cells for adoptive immunotherapy [19-24] as well.

Phenotypic analysis has often been utilized to characterize the differentiation, proliferation, activation status and immune response of a particular immune cell. Since there was no prior information about the phenotypic characteristics of lung tumor derived, tumor antigen-specific CD8^+ ^CTLs, we needed to first characterize the cell surface markers of the patient derived and *in vitro*-expanded CTLs. A previous study, which investigated the functions of CD8^+ ^T cell CD27^+^CD45RA^+ ^subsets, suggested that CD27^+^CD45RA^+^CD8^+ ^T cells are naïve T cells, while CD27^+^CD45RA^-^CD8^+^, CD27^-^CD45RA^-^CD8^+^, and CD27^-^CD45RA^+^CD8^+ ^T cells are memory, memory/effector and effector cells, respectively [44]. Classification is made on virus epitope specific CD8+ T cells based upon CCR5 and CCR7 expression indicates that CCR5 expression is restricted to memory and effector cells, whereas CCR7 is expressed only in naïve and memory cells [45]. The information we gathered after phenotypic analysis of CTLs revealed that these CD8^+ ^T cells were CD25^+^CD27^-^CD45RA^-^CD45RO^+^CD44^+^CD69^+ ^CCR5^+^CCR7^+^, and based on the previous studies [45] we can conclude that these cells have an activated memory phenotype, again suggesting that within the patient, positive immune stimulation took place at some point in the course of the disease. Yet, for many patients, and for reasons which are not yet clear, these cells fail to control tumor growth.

It has been difficult to study the antitumor efficacy of human immune effector cells, such as cytolytic T lymphocytes (CTLs) in the clinical setting of lung cancer and there has been little information derived from animal models in this regard and the models that have been developed utilizes subcutaneously grown tumors [5,46]. SCID mice that lack functional T and B cells have been shown to be useful hosts to grow human tumors, both from established cell lines and from tumors obtained directly from patients [46-53]. Thus, they may provide an environment for studying the interactions between human tumor cells and autologous tumor antigen-specific CTL. In evaluating the efficacy of these tumor antigen-specific CTLs *in vivo *by adoptively transferring them to SCID mice, we found that these CTLs possessed remarkable efficiency at homing to and remaining in the lung bearing autologous tumors (orthotopically grown tumors). Notably, they did not persist in non-tumor bearing lungs or liver. Most importantly, a single injection of these cells, with no further supportive treatments with IL-2 resulted in significant tumor growth suppression in both subcutaneously grown tumor and lung tumor metastasis. Future studies should evaluate other cytokines such as IL-15 in terms of improving CTL effectiveness.

One possibility for improvement of the response may be related to timing of immune treatment since based on our results, the scheduling of CTL injection was found to be very critical in delaying tumor growth. If tumor antigen-specific CTLs are generated early enough they may be more effective in controlling tumor growth. Another obvious reason for incomplete cure could be the limited survival of CTLs *in vivo*. Improving the survival of CTLs in this setting remains for further investigation. The number of CTLs is another issue that needs to be considered; in all our experiments we administered a maximum dose of 5 × 10^6 ^CTLs. In future experiments, it is essential to know whether the tumor growth will be further inhibited by using a higher dose of CTLs. Notably however, administration of CTLs at consecutive time points did not significantly enhance the antitumor effect compared to a single injection of CTLs at an early time point. This may be due to the fact that the first injection resulted in the majority of the effect and that possible tumor cell phenotype changes occurring after the first CTL treatment provided tumor cells with escape mechanisms from recognition and destruction by subsequently administered CTLs.

Any effective CD8+ T cell based-immunotherapy depends on the continued HLA class I antigen expression by tumor cells [7,54]. Although loss of HLA class I antigen expression is previously described in patient tumors [7], this study is the first study to show patients' HLA Class I positive lung tumor cells following tumor antigen-specific CTL treatment showed marked decreased HLA class I expression using this mouse model. Whether this change represents a general phenomenon is not known at present. Nevertheless our results suggest that the application of T cell selective pressure to a tumor cell population favours the outgrowth of tumor cells with defects in HLA class I antigen expression. At this point, we cannot distinguish between selection and ongoing mutation as the major mechanism for this effect. However, Kedl et al [55] have shown down-modulating peptide MHC class I complexes on APCs by high affinity T cells *in vivo*. Since we were still able to detect cytoplasmic but not membrane staining with anti-HLA class I mAb in some of the tumor cells, it is possible that a similar phenomena might be occurring after tumor antigen-specific CTL interaction with autologous tumor cells. In this study, we have not been able to look at changes in tumor antigen expression because we are still in the process of characterizing tumor antigen-specific antibodies. The mechanism(s) underlying decreased HLA class I expression on lung tumor cells is not known. The phenotype of the lung tumor cells we have analyzed in conjunction with the described high frequency of antigen processing machinery abnormalities in tumor cells suggest that defects in this machinery may underlie the HLA class I antigen downregulation we have detected in tumor cells following treatment with tumor antigen-specific CTL. If our hypothesis is correct, the defects in the antigen processing machinery which result in defective loading of HLA class I antigens with peptides and in their instability on the cell membrane may be corrected by IFN-γ. Another explanation of decreased MHC class I expression can be the selection of MHC class I negative variants of the tumor. The concept of selection for tumor cells or shaping the tumor immunogenicity has been previously described [56]. Using this model, ways could be tested to evaluate the ability of various adjuvants for their ability to increase expression of MHC class I. For example, tumor cells have been shown to upregulate their MHC class I expression after IFN-γ treatment [56-58]. An alternative strategy could rely on the enhancement of the lytic activity of natural killer (NK) cells, since HLA class I antigen downregulation and loss enhance susceptibility of target cells to NK cell-mediated lysis.

## Conclusion

These studies confirm and extend previous studies and demonstrate the ability of a patient's lung-tumor derived CTLs to specifically home to a orthotopically grown xenograft composed of the autologous tumor cells, suppress tumor growth and express IFN-γ *in vivo *without the administration of exogenous human cytokines. Moreover, these study shows killing of lung tumors mediated by CTLs can shape the phenotype of the tumors and result in tumor growth with decreased HLA Class I expression. Although caution should be exercised in interpreting these data since they derive from only one patient's lung tumor, the approach developed here could be used for additional patients and thus contribute to our understanding of the potential of adoptively transferred CTLs in the immunological control of tumor growth. Lastly, these studies provide hope that additional work maximizing the long-term effectiveness of this type of treatment will provide important clues toward development of improved adoptive immunotherapy strategies for humans with lung cancer.
